# Distributed Artificial Intelligence Models for Knowledge Discovery in Bioinformatics

**DOI:** 10.1155/2015/846785

**Published:** 2015-03-25

**Authors:** Juan M. Corchado, Isabelle Bichindaritz, Juan F. De Paz

**Affiliations:** ^1^Biomedical Research Institute of Salamanca/BISITE Research Group, University of Salamanca, Edificio I+D+i, 37008 Salamanca, Spain; ^2^Computer Science Department, State University of New York, Shineman Center 427, Oswego, NY 13126, USA

The increasing volume of existing information on biological processes and the use of large databases have significantly increased the accessibility of datasets to the scientific community. This has enabled performing an analysis to facilitate the extraction of relevant information or modeling and optimizing tasks in different processes. Parallel to the increasing volumes of information is the emergence of new or adapted distributed computing models such as grid computing and cloud computing. Together with new techniques of artificial intelligence, or more specifically knowledge discovery, these management systems are making it possible to perform a more efficient analysis of the information and are enabling the creation of adaptive systems with learning ability.

In the area of distributed artificial intelligence models for knowledge discovery in bioinformatics, ten interesting proposals are presented. These models analyze different biological aspects and simulate the process or user behavior in the health care system. The main characteristics in these proposals are the use of artificial intelligence techniques to analyze the information and extract knowledge.

“Bladder Carcinoma Data with Clinical Risk Factors and Molecular Markers: A Cluster Analysis” provides interesting research about bladder cancer. The paper shows the hypothesis that the use of clinical and histopathological data with information about marker is useful to manage treatments of nonmuscle invasive bladder cancers (NMIBC). The authors apply data mining techniques such as hierarchical clustering to create molecular cluster and risk groups. In their experiments, the authors analyze 45 patients with a new diagnosis of NMIBC. They create four groups of patients and categorize the patients according to clinical characters and biological behavior.

The authors of “A Linear-RBF Multikernel SVM to Classify Big Text Corpora” use data mining techniques based on classifiers to big text corpora. In particular, they implement a variant of support vector machine (SVM) to reduce the computational cost. The authors show a multikernel SVM with automatic parameterization to improve the results of SVM parameterized under a brute force search. The proposal is composed of a workflow with algorithms to process documents, reduce the dimensionality of the data, and to apply/provide clustering, training, and prediction. The proposal is analyzed according to the classification results and building time in the dataset TREC Genomics 2005 corpus.

In “Analysis of Environmental Stress Factors Using an Artificial Growth System and Plant Fitness Optimization,” the authors analyze how some environment conditions can accelerate the evolution process; in addition to modifying the environment conditions, it is possible to select genomic variants. The authors analyze several factors for* Pleurotus ostreatus* to improve quality and production. In order to carry out the analysis of the factors, the authors include several IoT sensors to retrieve the information from the sensors. The information is then processed with data mining techniques in cloud system in order to predict plant growth and manage plant growth control.

“RecRWR: A Recursive Random Walk Method for Improved Identification of Diseases” proposes a new method to select the best disease targets for multiconcepts graphs. The proposal, referred to as recursive random walk with restarts (RecRWR), includes multilayer networks, graphs, and weights calculation and has been tested with the OMIM database with the area under ROC curves.

“Gene Knockout Identification Using an Extension of Bees Hill Flux Balance Analysis” is an interesting paper that presents an extension of Bees Hill Flux Balance Analysis (BHFBA) integrated within the framework of the OptKnock and Hill Climbing algorithm in a local search to extract gene knockout in order to maximize the production of the desired metabolite. The proposal has been analyzed with different databases and compares the execution time, growth rate, and production yield. The authors validate that the BHFBA improves the performance due to the inclusion of the Hill Climbing algorithm.

In “Using the eServices Platform for Detecting Behavior Patterns Deviation in the Elderly Assisted Living: A Case Study,” the authors present the eServices platform (Elderly Support Service Platform). The platform detects any deviation in the behavioral pattern followed by elderly people and is able to predict dangerous situations. The system was modeled with the CRISP-DM methodology and was tested with synthetic data based on real data and expert knowledge. The authors incorporate several data mining techniques such as decision trees, cluster, or ROC curves in order to validate the results.

“Agent-Based Spatiotemporal Simulation of Biomolecular Systems within the Open Source MASON Framework” presents a tool for biomolecular reaction modelling in the multiagent simulator of neighborhoods framework (MASON). The tool describes the interaction among the molecules. The system is worth considering to analyze the effect of different factors in simulations. The system is tested in several scenarios based on enzymatic activity. The results area is compared with studies performed in laboratories.

“A Distributed Multiagent System Architecture for Body Area Networks Applied to Healthcare Monitoring” presents an architecture based on a multiagent system to monitor the parameters of several users with biomedical sensors. The agents incorporate the BDI (belief, desire, and intention) model and data mining techniques to analyze the data of the sensors. The system is able to detect movements, activities, and postures. Its accuracy is confirmed and presented in the results taken from the tests performed in several cases.

“Modelling the Longevity of Dental Restorations by means of a CBR System” presents a case based reasoning (CBR) system to analyze dental restorations with different materials. The system applies the steps of the CBR cycle in which it integrates several data mining algorithms to perform in the retrieved phase. It also includes a mixture of experts with neural networks and Bayesian networks in the reuse phase to predict the longevity of dental restorations. The system is tested with the data of patients and the results are shown in the paper.

Finally, “aCGH-MAS: Analysis of aCGH by means of Multiagent System” proposes a multiagent system to analyze data of CGH (comparative genomic hybridization) arrays. The agents incorporate several layers to perform visualization and analysis and to manage information processes. The system proposes different visualizations to facilitate the visual analysis and to access the information in databases. Moreover, the multiagent system provides several data mining techniques to carry out the analysis. The system is tested with several kinds of CGH arrays and the results are shown in the paper.

